# Umbilical cord-matrix stem cells induce the functional restoration of vascular endothelial cells and enhance skin wound healing in diabetic mice via the polarized macrophages

**DOI:** 10.1186/s13287-020-1561-x

**Published:** 2020-01-28

**Authors:** Shichang Zhang, Li Chen, Guoying Zhang, Bo Zhang

**Affiliations:** 1 Department of Laboratory Medicine, the First Affiliated Hospital of Nanjing Medical University, Nanjing, 210029 China; 2 Department 4, State Key Laboratory of Trauma, Burns and Combined Injury, Institute of Surgery Research, Daping Hospital, Army Medical University, Chongqing, 400042 China; 3 Department of Obstetrics, the First Affiliated Hospital of Nanjing Medical University, Nanjing, 210029 China

**Keywords:** Umbilical cord-derived matrix stem cells, Wound healing, Macrophages, Vascular endothelial cells, Diabetes mellitus

## Abstract

**Background:**

Chronic nonhealing wounds represent one of the most common complications of diabetes and require advanced treatment strategies. Increasing evidence supports the important role of mesenchymal stem cells in diabetic wound healing; however, the underlying mechanism remains unclear. Here, we explored the effects of umbilical cord-matrix stem cells (UCMSCs) on diabetic wound healing and the underlying mechanism.

**Methods:**

UCMSCs or conditioned medium (UCMSC-CM) were injected into the cutaneous wounds of streptozotocin-induced diabetic mice. The effects of this treatment on macrophages and diabetic vascular endothelial cells were investigated in vivo and in vitro.

**Results:**

Our results reveal that UCMSCs or UCMSC-CM accelerated wound healing by enhancing angiogenesis. The number of host macrophages recruited to the wound tissue by local infusion of UCMSCs was greater than that recruited by fibroblast transplantation or control. The frequency of M2 macrophages was increased by UCMSC transplantation or UCMSC-CM injection, which promoted the expression of cytokines derived from M2 macrophages. Furthermore, when cocultured with UCMSCs or UCMSC-CM, lipopolysaccharide-induced macrophages acquired an anti-inflammatory M2 phenotype characterized by the increased secretion of the cytokines interleukin (IL)-10 and vascular endothelial growth factor and the suppressed production of tumor necrosis factor-α and IL-6. UCMSC-CM-activated macrophages significantly enhanced diabetic vascular endothelial cell functions, including angiogenesis, migration, and chemotaxis. Moreover, the action of UCMSC-CM on macrophages or vascular endothelial cells was abrogated by the administration of neutralizing antibodies against prostaglandin E2 (PGE2) or by the inhibition of PGE2 secretion from UCMSCs.

**Conclusions:**

Our findings demonstrate that UCMSCs can induce the functional restoration of vascular endothelial cells via the remodeling of macrophage phenotypes, which might contribute to the marked acceleration of wound healing in diabetic mice.

**Graphical Abstract:**

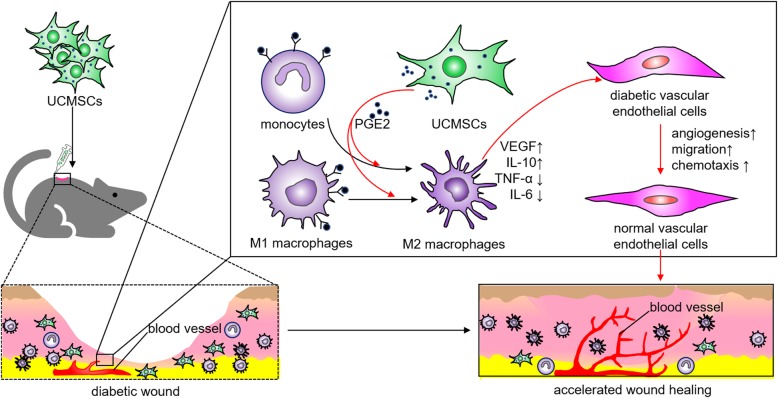

## Introduction

Chronic wounds, such as diabetic ulcers, are a major clinical problem in diabetic patients and can lead to severe outcomes, including increased amputation rates and even death [1]. Despite the availability of clinical treatments for chronic wounds, the amputation rate is high. Among the many factors contributing to diabetic ulcers, impaired angiogenesis by endothelial cell dysfunction is crucial [2]. There is growing evidence suggesting that mesenchymal stem cell (MSC)-based therapies can promote diabetic wound healing via enhanced angiogenesis, which obviates the distress of nonhealing and amputation [2–5]. Therefore, MSC transplantation offers a novel treatment strategy to augment diabetic wound healing [2, 6].

MSCs derived from autologous and allogeneic tissues have been used for the cell therapy of diabetic ulcers [2, 3]. The therapeutic potential of autologous stem cells is impaired by the severe reduction in number and dysfunction of MSCs derived from diabetic patients with long-term disease [7, 8]. Previous studies have indicated that the number and function of MSCs decrease with age. The majority of diabetic patients are elderly and consequently lack sufficient numbers of autologous stem cells for the cell therapy of diabetic ulcers [9, 10]. Among the available sources of MSCs, the umbilical cord represents a cost-effective, productive, feasible, acceptable, and universal source from which to isolate MSCs [11]. Although umbilical cord-matrix stem cell (UCMSC) transplantation has been shown to promote wound healing, there have been few reports in diabetes mellitus [12–15]. The underlying mechanism of the UCMSC-induced acceleration of diabetic wound healing remains unclear.

Increasing evidence has demonstrated that transplanted MSCs can act as an initiator to trigger endogenous cell-mediated tissue repair [16–19]. The repair of cutaneous wounds is a highly complex biological process involving several cell types, cytokines, and extracellular matrix components [20]. Angiogenesis is a central process in wound healing and impaired angiogenesis is a feature of diabetic wounds [21]. The dysfunction of vascular endothelial cells contributes to poor wound healing in diabetes [21]. Diabetic wound healing and angiogenesis are enhanced by MSCs derived from different sources [4, 22, 23], which suggests that the recovery of vascular endothelial cell dysfunction is initiated by MSCs. However, the precise mechanisms by which MSCs correct vascular endothelial cell dysfunction are still unclear.

The therapeutic effects of MSCs might be attributable to their ability to stimulate the survival and functional recovery of resident cells or to regulate the local microenvironment or niche and immune response [24]. Macrophages are a fundamental cell type in wound healing and immunity. M1- and M2-polarized macrophages are key components of tissue repair and remodeling that occur during wound healing [25]. Therefore, UCMSCs may induce the restoration of vascular endothelial cell dysfunctions via the remodeling of the macrophage phenotypes during wound healing in diabetes.

In this study, the therapeutic potential of UCMSCs on the full-thickness excisional skin wounds of diabetic mice was investigated. In addition, the effects of UCMSCs on the polarization of macrophages were examined in both in vivo and in vitro experiments. Furthermore, the role of UCMSC-polarized macrophages in the restoration of vascular endothelial cell dysfunction, which was induced by high glucose, was identified. Finally, the mechanisms by which UCMSCs enhance cutaneous wound healing in diabetic mice were elucidated. These findings will promote the clinical application of MSC-based therapy for diabetic ulcers.

## Materials and methods

### Tissue sources and cell culture

Umbilical cords were obtained from full-term delivered infants. Normal skin was obtained from healthy donors undergoing plastic surgery procedures (mostly dermolipectomies), both male and female, excluding pregnant women. Tissues were obtained at the Department of Obstetrics and Gynecology and the Department of Plastic Surgery, respectively, at the First Affiliated Hospital of Nanjing Medical University. Tissue specimens were collected following the principles of the Declaration of Helsinki. This study was approved by the Research Ethics Committee of the First Affiliated Hospital of Nanjing Medical University, and all participants provided informed written consent.

The isolation, expansion, and characterization of UCMSCs were performed as described in our previous study [26]. Primary human normal skin fibroblasts (FBs) were obtained from skin tissue samples by the collagenase digestion method [27] and were cultured in Dulbecco’s modified Eagle’s medium (DMEM, Gibco, Grand Island, USA) as previously described [28]. Human umbilical vein endothelial cells (HUVECs) were isolated as previously described [29] and cultured in epithelial growth medium (EGM)-2 basal medium supplemented with 2% fetal bovine serum (FBS) and a EGM-2 SingleQuot Kit (Lonza, Basel, Switzerland). In all experiments, passages 2–4 were tested. Raw264.7 mouse monocytes/macrophages (from the American Type Culture Collection) were cultured in high-glucose DMEM containing 10% FBS (Gibco), 100 units/mL penicillin, and 100 μg/mL streptomycin.

### UCMSC-conditioned medium (UCMSC-CM) production

UCMSCs at passages 3–4 (P3-P4) were grown to 80% confluence in DMEM supplemented with 10% knockout serum replacement and 2 mM l-glutamine (Gibco). The UCMSC-CM was collected 24 h later and concentrated fivefold using ultrafiltration units with a 3-kDa cutoff (Millipore, Bedford, USA). The concentrated CM was immediately cryo-preserved at − 80 °C until use. Nonconditioned medium (NCM) without UCMSCs was used as a control.

To confirm the role of Prostaglandin E2 (PGE2), the effects of inhibitors of cyclooxygenase-2 (an essential enzyme in PGE2 synthesis) on UCMSCs were investigated. UCMSCs were treated with indomethacin (1 μM, Sigma-Aldrich, St. Louis, USA) or NS-398 (1 μM, Sigma-Aldrich) for the preparation of indomethacin-CM or NS-398-CM, respectively.

### Diabetic mouse skin wound model and treatments

Forty-eight C57BL/6J female mice (age 8–10 weeks and weighing 20–25 g) were purchased from the Laboratory Animal Center, Army Medical University. Obliteration of pancreatic β cells was achieved with the intraperitoneal injection of 60 mg/kg streptozotocin (STZ; Sigma-Aldrich) in 50 mM sodium citrate buffer (pH 4.5) for 5 consecutive days. Ten days after the initial injection, mice with a blood glucose level > 300 mg/dL were deemed diabetic, whereas those with a level < 300 mg/dL received an additional 3 days of STZ injection (50 mg/kg). Mice were considered diabetic if their glucose levels remained > 300 mg/dL for at least 4 weeks before the date of wounding. After shaving the mice, two full-thickness excisional skin wounds (8 mm in diameter) were created on the upper dorsal surface of each mouse. Then, the mice were randomly divided into four treatment groups: (1) control group (wounds treated with 150 μL NCM), (2) FB group (wounds treated with 1 × 10^6^ FBs in 150 μL PBS), (3) UCMSC group (wounds treated with 1 × 10^6^ UCMSCs in 150 μL PBS), and (4) UCMSC-CM group (wounds treated with 150 μL UCMSC-CM). Briefly, the mice were subcutaneously injected with FBs, UCMSCs, UCMSC-CM, or PBS around the wounds at six injection sites (25 μL per site). Each mouse in the UCMSC-CM group received an injection of 150 μL every day for three consecutive days. At days 0, 3, 7, and 14 post wounding, the wound surface area was recorded, and digital photographs were obtained using an Olympus digital camera. The wound area was quantified using ImageJ software (NIH, Bethesda, MD) and was expressed as the percentage of original wound size over time.

For in vivo tracing, the UCMSCs were prelabeled with the membrane dye PKH26 according to the instructions provided by the manufacturer (Sigma-Aldrich). In separate experiments, PKH-26-labeled UCMSCs were transplanted into wounds of 9 diabetic mice. Wound bed tissues of diabetic mice were harvested at 1, 7, and 14 days after UCMSC transplantation. These tissues were placed into optimal cutting temperature (OCT) compound (Sakura Finetek, Tokyo, Japan) and cut into 4-μm-thick sections on a cryostat. All nuclei in the sections were stained with 4,6-diamidino-2-phenylindole (DAPI; Sigma-Aldrich). PKH26-labeled UCMSCs in wound bed tissues were detected by fluorescence microscopy (Olympus, Tokyo, Japan).

### Histological analysis and immunofluorescence analysis

Wound bed tissues from each group were harvested at 14 days post administration and were fixed with 4% paraformaldehyde. The samples were then embedded in paraffin, and 4-μm sections were used for hematoxylin and eosin (H&E) staining. Six pictures of each specimen slice were captured. The capillary-like structures were analyzed with ImageJ software.

Wound bed tissues of each group were harvested at 3 days post administration and were divided into two parts along their central axis. One half was fixed with 4% paraformaldehyde and was embedded in OCT compound and cut into 4-μm-thick sections on a cryostat. The other half was used for RNA extraction. The sections were then permeabilized with 0.1% (v/v) Triton X-100 in PBS for 20 min, blocked with 5% (v/v) bovine serum albumin for 30 min, and incubated overnight with the following primary antibodies purchased from Abcam (Cambridge, UK): anti-F4/80 (rat IgG, 1:200), anti-Ly6C (rat IgG, 1:200), and anti-CD206 (mouse IgG, 1:400). The following secondary antibodies were purchased from Invitrogen: anti-rat IgG-TRITC and anti-mouse IgG-FITC. After counterstaining with DAPI, tissue images were captured with a Leica confocal microscopy system. The average number of F4/80-, Ly6C-, CD206-, and F4/80-positive cells was determined by counting 45 random fields under universal fluorescence microscopy; at least 6 animals per group were examined.

### Quantitative real-time PCR

Total RNA was extracted from wound bed tissues acquired 3 days post administration using a RNeasy Mini kit (Qiagen, Inc., Valencia, CA) according to the manufacturer’s instructions. The total RNA concentration was determined by optical density at 260 nm using a spectrophotometer. The reverse transcription reaction from 1 μg of RNA template was carried out using a First Strand cDNA Synthesis kit (TOYOBO, Japan). Quantitative real-time PCR was performed using SYBR Green Real-time PCR Master Mix (TOYOBO) and detected by a LightCycler 480 II real-time PCR system (Roche, USA). The expression level was analyzed and normalized to GAPDH in the cDNA samples. The fold change in gene expression was calculated using the 2-ΔΔCT method. Primer sequences are provided in Additional file 1: Table S1.

### In vitro effects of UCMSCs on macrophages

To investigate the effects of UCMSCs on macrophages, Raw264.7 cells were suspended in a culture medium and stimulated with 100 ng/mL lipopolysaccharide (LPS; Sigma-Aldrich). After 5–10 min, 1 × 10^5^ LPS-induced macrophages were transferred to 6-well culture plates and treated as follows: (1) cocultured with 1 × 10^5^ UCMSCs, which were seeded onto the 0.4-μm-pore-size Corning Transwell inserts; (2) cultured in concentrated UCMSC-CM diluted 1:4 in fresh medium; and (3) cultured in fresh medium as a control. After 3 days, supernatants were collected, centrifuged to remove possible cell contamination (500×*g* for 10 min), and stored at − 20 °C until the levels of cytokines were examined by enzyme-linked immunosorbent assay (ELISA).

### In vitro angiogenesis assays

Subconfluent HUVECs were harvested with trypsin/EDTA, seeded into 6-well plates at 4 × 10^5^ cells/well, and incubated overnight to allow adhesion. Adherent cells were then incubated under high-glucose concentration (30 mM) conditions in EGM-2 for 72 h.

Subconfluent HUVECs were incubated overnight in EGM-2 plus 2% FBS containing NCM, UCMSC-CM diluted 1:4, or cocultured with LPS-treated macrophages or UCMSC-CM-treated macrophages. These HUVECs were detached with trypsin/EDTA and resuspended in EBM-2 plus 0.1% FBS containing NCM, UCMSC-CM diluted 1:4, or cocultured with LPS-treated macrophages or UCMSC-CM-treated macrophages. The formation of network structures was assessed using the reduced growth factor Matrigel™ (BD Biosciences) thick gel method according to the manufacturer’s instructions. HUVECs were seeded at 3 × 10^4^ cells/well in 6-well slide chambers in 100 μL of Matrigel. The chambers were incubated under the aforementioned four conditions at 37 °C and 5% CO_2_ overnight. The wells were then photographed under phase-contrast inverted microscopy at × 4 and × 10 magnification. For each condition, network extension was measured using the ImageJ software, as previously described [30]. Each condition was tested in sextuplicate, and the assay was repeated twice.

### In vitro migration assays

The ability of UCMSCs to stimulate HUVEC migration was evaluated in the scratch assay. HUVECs grown to form a confluent monolayer in 100 μg/mL fibronectin-coated 6-well plates were starved in EBM-2 containing 0.1% FBS under high-glucose concentration (30 mM) conditions for 24 h. A central scratch was created by scraping cells away with a 200-μL pipette tip. After the removal of debris by washing the cells with PBS, cells were incubated with EBM-2 containing 2 mM hydroxyurea (Sigma-Aldrich) to induce growth arrest in the presence of NCM, UCMSC-CM diluted 1:4, or cocultured with LPS-treated macrophages or UCMSC-CM-treated macrophages. Before incubation and after 24 h of incubation, cells were washed. Scratches were photographed at × 4 magnification at 25%, 50%, and 75% of the scratch length and distance. The scratch area was measured using the ImageJ software before and after incubation. Each condition was tested in sextuplicate, and the assay was repeated six times.

### In vitro chemotaxis assays

HUVEC chemotaxis towards UCMSC-CM diluted 1:4 or in cocultures with LPS-treated macrophages or UCMSC-CM-treated macrophages was assayed with a modified 96-well Boyden chamber (Neuro Probe) using an 8-μm-pore-size membrane from the Transwell system. The membrane of the Boyden chamber was precoated with type I collagen (10 μg/mL in PBS, Sigma-Aldrich) at room temperature for 1 h overnight and then washed with PBS. Subconfluent HUVECs were starved in EBM-2, respectively, under high-glucose (30 mM) conditions for 24 h. HUVECs were detached with 0.02% PBS/EDTA, resuspended in EBM-2, and then placed in the upper chamber at a concentration of 5000 cells/well. The stimuli (NCM, UCMSC-CM diluted 1:4, or coculture with LPS-treated macrophages or UCMSC-CM-treated macrophages) were added to the lower chamber. During all the starvation and experimental period, HUVECs were incubated in EBM-2 containing 0.1% FBS. The chambers were maintained at 37 °C and 5% CO_2_ for 4 h. The upper surface-adherent cells were removed by scrapping, and the lower surface-adherent cells were considered to have migrated. The membranes were fixed with 100% methanol at room temperature for 5 min and then mounted on glass slides with medium containing DAPI. The numbers of stained nuclei were counted in five high-power fields per well under fluorescence microscopy.

### Identification of the effect of UCMSC-secreted PGE2 on LPS-treated macrophages

To determine the involvement of UCMSC-secreted PGE2 in UCMSC-CM-induced macrophage polarization, LPS-treated macrophages were stimulated with NS-398-CM or a mixture of UCMSC-CM with an anti-PGE2 neutralizing antibody (10 μg/mL, Cayman Chemical). After 3 days, the supernatants were collected and stored at − 20 °C until use. In addition, NCM was mixed with PGE2 (2 ng/mL, Sigma-Aldrich) prior to the addition of the mixture to stimulate LPS-treated macrophages as a positive control. The cytokine levels of these supernatants were examined by ELISA.

### Evaluation of the role of PGE2 in promoting the UCMSC-mediated functional recovery of diabetic vascular endothelial cells via LPS-treated macrophages

The involvement of UCMSC-secreted PGE2 in the effects of UCMSCs on the functional assays of high-glucose-treated HUVECs via macrophages was evaluated by angiogenesis, migration, and chemotaxis assays. HUVECs cocultured with LPS-treated macrophages were treated with NS-398-CM, a mixture of UCMSC-CM with anti-PGE2 neutralizing antibody, or NCM plus PGE2.

### Enzyme-linked immunosorbent assay (ELISA)

The levels of PGE2, tumor necrosis factor (TNF)-α, interleukin (IL)-6, IL-10, and vascular endothelial growth factor (VEGF) in the supernatants of cultured cells were determined using ELISA kits (R&D Systems, Minneapolis, MN) according to the manufacturer’s protocols. The concentrations of TNF-a, IL-6, IL-10, and VEGF were normalized to the total protein content.

### Statistical analysis

Data are presented as the means ± standard deviation of *n* determinations as indicated in the figure legends. Statistical analysis was performed with GraphPad Prism 7 software (GraphPad, San Diego, CA). One-way ANOVA with Tukey’s multiple comparisons test was used to compare each parameter when there were two or more independent groups. A two-way ANOVA of grouped analyses was used to compare the wound recovery rate at different time points, and then Bonferroni post hoc *t* tests were performed to compare replicate means by row when the ANOVA was statistically significant. Values of *P* < 0.05 were considered statistically significant.

## Results

### UCMSCs and UCMSC-CM treatment accelerated the rate of wound healing in diabetic mice

**Fig. 1:**
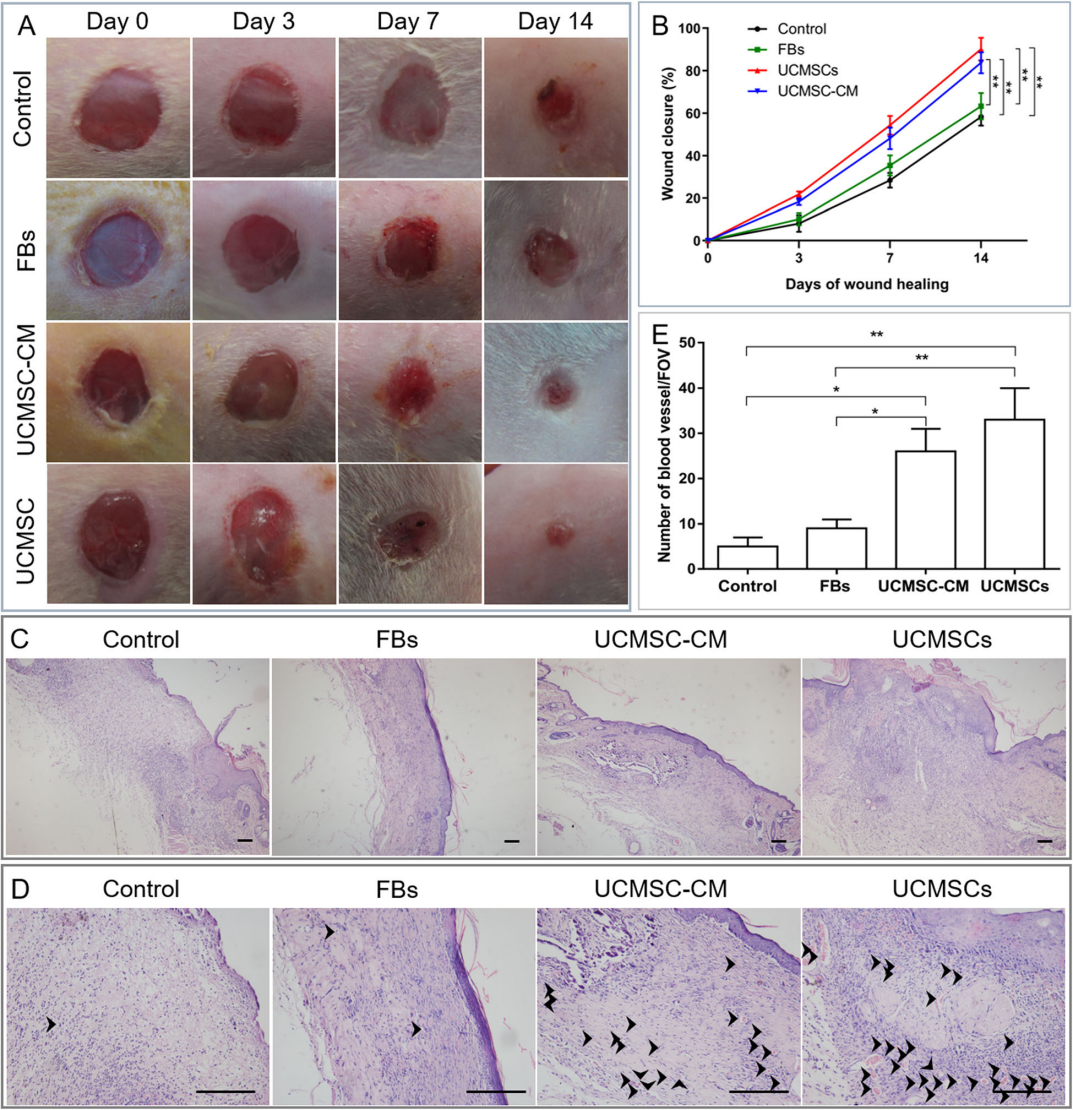
UCMSCs or UCMSC-CM accelerates skin wound healing in diabetic mice. **a** Gross view of wounds treated with PBS, FBs, UCMSCs, or UCMSC-CM at days 0, 3, 7, and 14 post administration. **b** The rate of wound closure in wounds receiving different treatments at the indicated times. **c** Histological images (H&E staining) of wound sections treated with PBS, FBs, UCMSCs, or UCMSC-CM at day 14 post administration. Scale bar 100 μm. **d** Histological images of the capillary-like structures in wound bed tissues of different groups. Black arrowheads indicate the capillary-like structures. Scale bar 100 μm. **e** Quantitative analysis of capillary densities in wound bed tissue. *n* = 12 per group. *P < 0.05, **P < 0.01

To investigate the therapeutic potential of UCMSCs and UCMSC-CM on the wound healing of streptozotocin-induced diabetic mice, either UCMSCs or UCMSC-CM were administered to the local wound site. Figure 1a shows that the wound closure in UCMSC- or UCMSC-CM-treated mice was significantly accelerated compared with that of FB-treated mice or NCM-treated mice; the wound closure in FB-treated diabetic mice was not significantly improved compared with that in NCM-treated mice. The area under curve analysis revealed that the overall rate of wound healing was significantly greater in mice that received UCMSCs or UCMSC-CM starting from day 0 to day 14 than in mice that received FB or PBS administration (Fig. 1b). There were no differences in wound closure rate between UCMSC- and UCMSC-CM-treated mice. Histologic analysis showed that UCMSC or UCMSC-CM treatment accelerated wound healing by enhancing angiogenesis, re-epithelialization, and granulation tissue (Fig. 1c). Furthermore, a larger number of capillary-like structures were observed in the wound site of UCMSC- or UCMSC-CM-treated mice (Fig. 1c, d). Capillary densities in the wound site at 14 days posttreatment were assessed morphometrically after H&E staining. The density of blood vessels was significantly higher in the wound site of UCMSC- or UCMSC-CM-treated mice than in that of FB- or NCM-treated mice (Fig. 1e). However, the number of transplanted cells labeled with PKH26 in the wound tissue decreased gradually over time after cell transplantation (Additional file 1: Figure S1). These results suggest that UCMSC or UCMSC-CM administration can enhance wound healing and angiogenesis in diabetic mice.

### UCMSC and UCMSC-CM treatment increased the percentage of M2 macrophages in the local wounds of diabetic mice

**Fig. 2:**
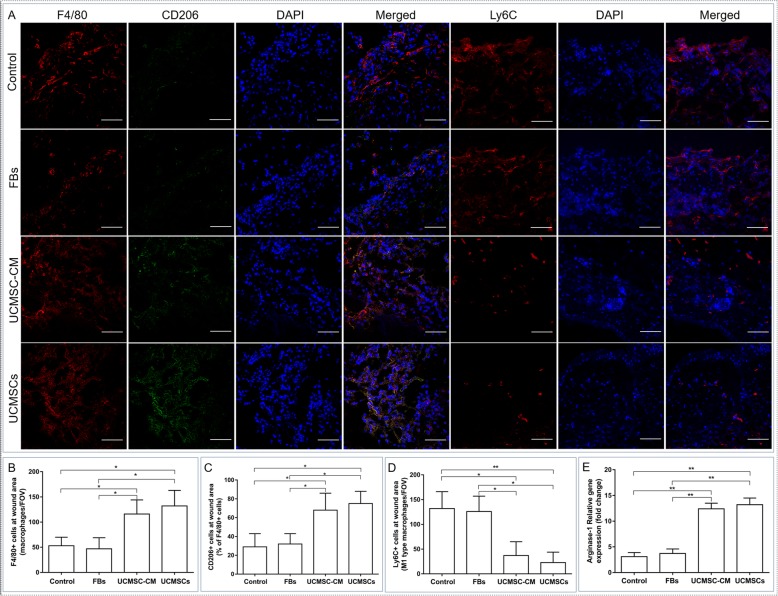
UCMSCs or UCMSC-CM increases the percentage of M2 macrophages in the local wounds of diabetic mice. **a** Frozen sections of wound bed tissues from diabetic mice at 3 days after treatment were immunostained with specific antibodies against F4/80, CD206, and Ly6C. Nuclei were stained with DAPI (blue). Scale bar 100 μm. **b** Frequency of F4/80-positive macrophages in wound bed tissue. **c** Frequency of CD206- and F4/80-positive macrophages in wound bed tissue. **d** Frequency of Ly6C-positive cells in wound bed tissue. **e** The level of arginase-1 expression in wound bed tissues. *n* = 6 per group. *P < 0.05, **P < 0.01

To evaluate the effects of transplanted UCMSCs on the phenotype of infiltrating macrophages at day 3 posttreatment, wound tissues were immunostained for F4/80, CD206 for M2 macrophages, and Ly6C. As shown in Fig. 2a, a large number of F4/80-positive macrophages were observed in the wound sites of diabetic mice. The number of F4/80- and CD206-positive macrophages in the wound site of UCMSC- or UCMSC-CM-treated mice was significantly higher than that of FB- or NCM-treated mice, whereas the number of Ly6C-positive cells in the wound site of UCMSC- or UCMSC-CM-treated mice was significantly lower than that of FB- or NCM-treated mice (Fig. 2a–d). Furthermore, the relative expression of arginase-1, which is another well-known marker for M2 macrophages, was higher in the wound site of UCMSC- or UCMSC-CM-treated than FB- or NCM-treated mice (Fig. 2e). These findings demonstrate that UCMSCs or UCMSC-CM can increase the percentage of M2 macrophages and decrease the number of Ly6C-positive cells in the local wound sites of diabetic mice, potentially contributing to the regulation of the inflammatory response and enhancing the healing of diabetic wounds.

**Fig. 3:**
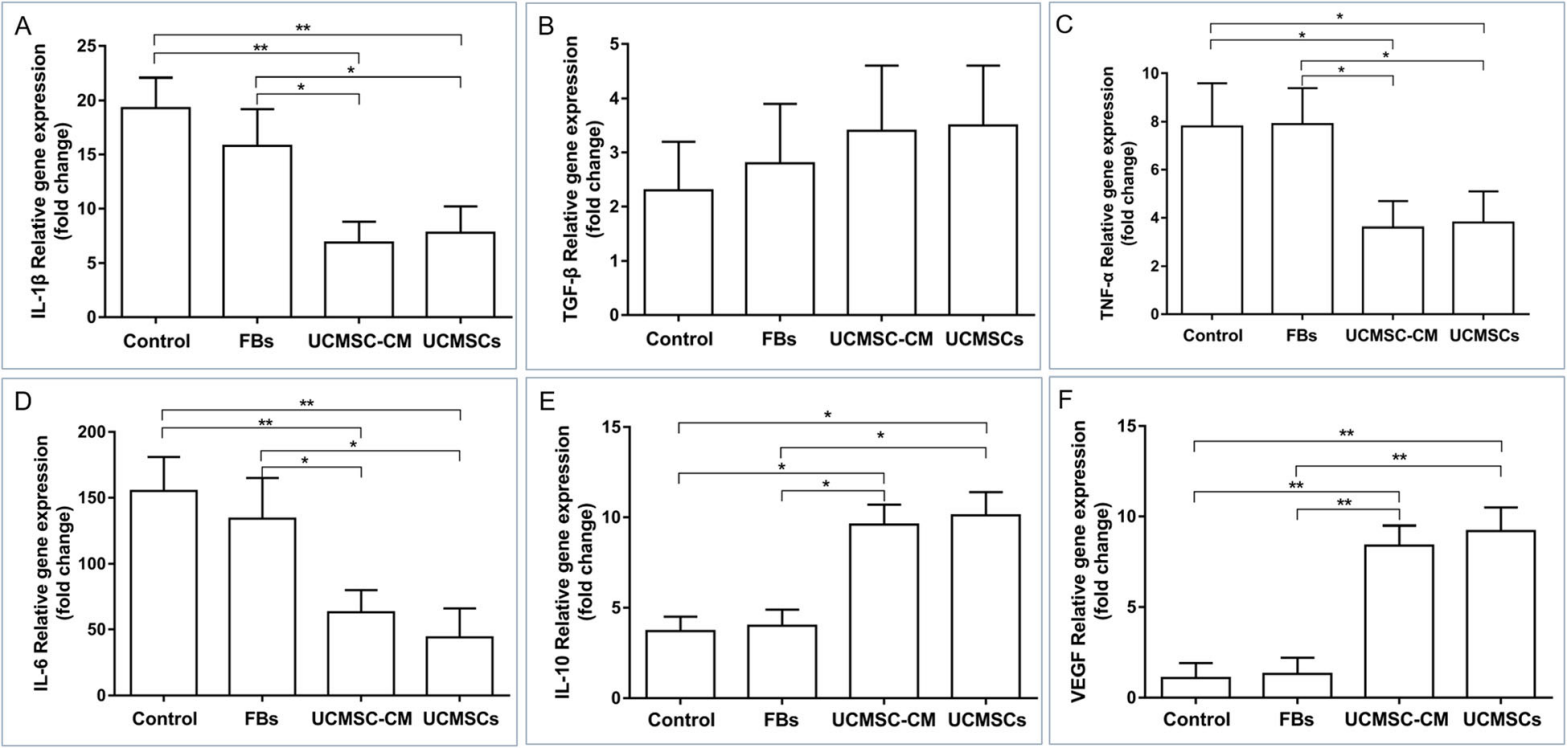
UCMSCs regulate the local inflammatory microenvironments of the wound bed tissues. Relative gene expression levels of several cytokines in the wound bed tissue at day 3 post treatment were quantified by real-time PCR. **a** IL-1β. **b** TGF-β. **c** TNF-α. **d** IL-6. **e** IL-10. **f** VEGF. *n* = 6 per group. *P < 0.05, **P < 0.01

The local inflammatory microenvironment of the wound tissues was further investigated. Real-time PCR results indicated that UCMSC or UCMSC-CM treatment significantly increased the local levels of both anti-inflammatory cytokines, IL-10 and VEGF, and significantly decreased the levels of proinflammatory cytokines, IL-1β, TNF-α, and IL-6 (Fig. 3a–f). These results further demonstrate that UCMSC or UCMSC-CM treatments promote diabetic wound healing, at least in part, by increasing the percentage of M2 macrophages, which regulate the local inflammatory microenvironment of the wound tissues.

### UCMSCs induced an M2 macrophage immune profile in coculture

**Fig. 4:**
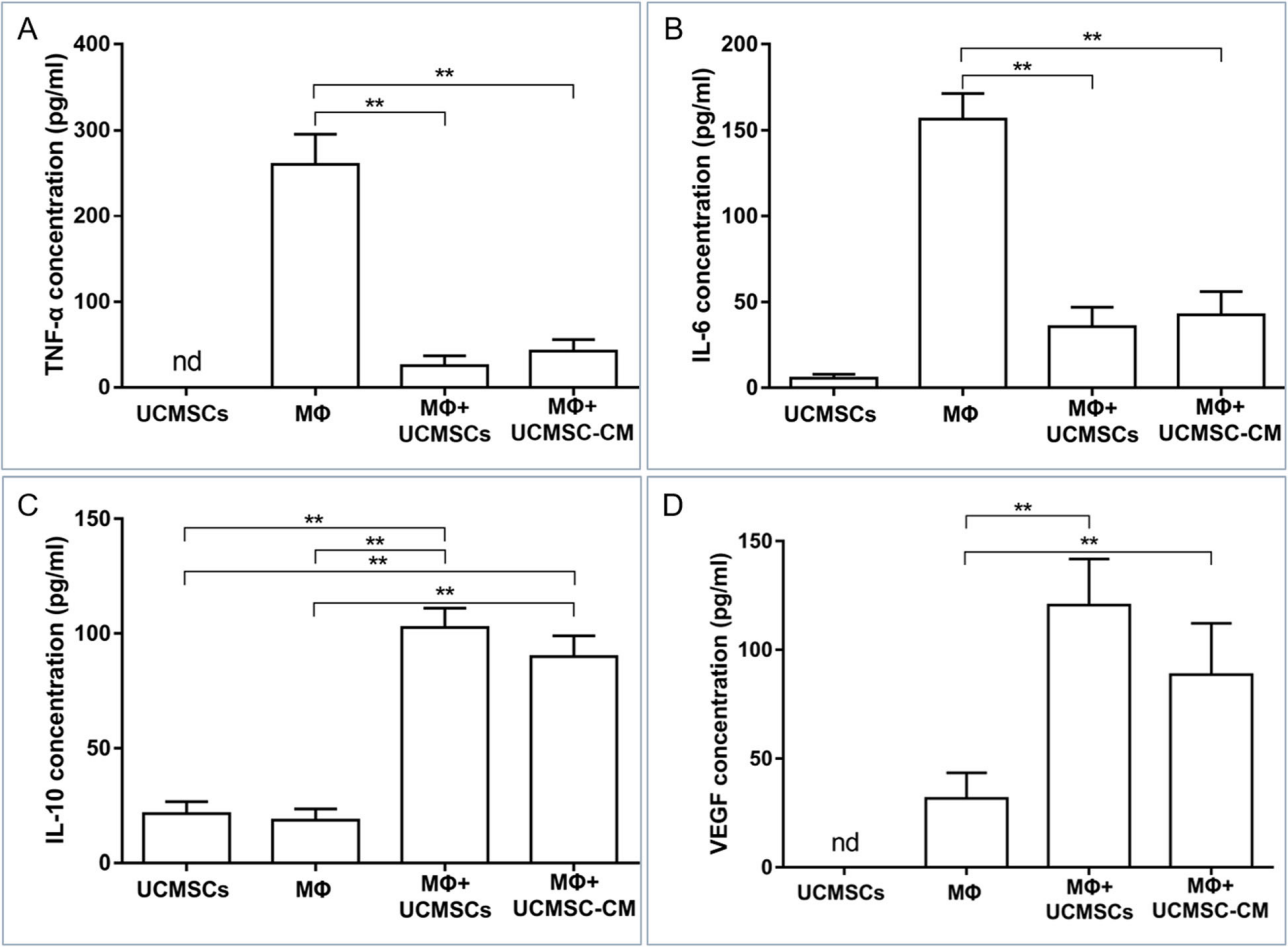
UCMSCs induced an M2 macrophage immune profile in coculture. The concentrations of several cytokines in the supernatants of LPS-treated macrophages, UCMSCs, or their cocultures were determined by ELISA, respectively. **a** TNF-α. **b** IL-6. **c** IL-10. **d** VEGF. *n* = 6 per group. *P < 0.05, **P < 0.01

The cytokine profile in the supernatants from LPS-induced macrophages, UCMSCs, or their cocultures was further determined by ELISA. The levels of TNF-α and IL-6 secreted by macrophages were decreased in the supernatants of UCMSC-cocultured macrophages or UCMSC-CM-treated macrophages (Fig. 4a, b). On the contrary, macrophages cocultured with UCMSCs or treated with UCMSC-CM produced significantly increased levels of IL-10 and VEGF in the supernatants (Fig. 4c, d). These results suggest that UCMSCs or UCMSC-CM are capable of switching macrophages from the M1 phenotype to the M2 phenotype.

### UCMSC-CM treatment rescued the diabetic vascular endothelial cell dysfunction via M2 macrophages

**Fig. 5:**
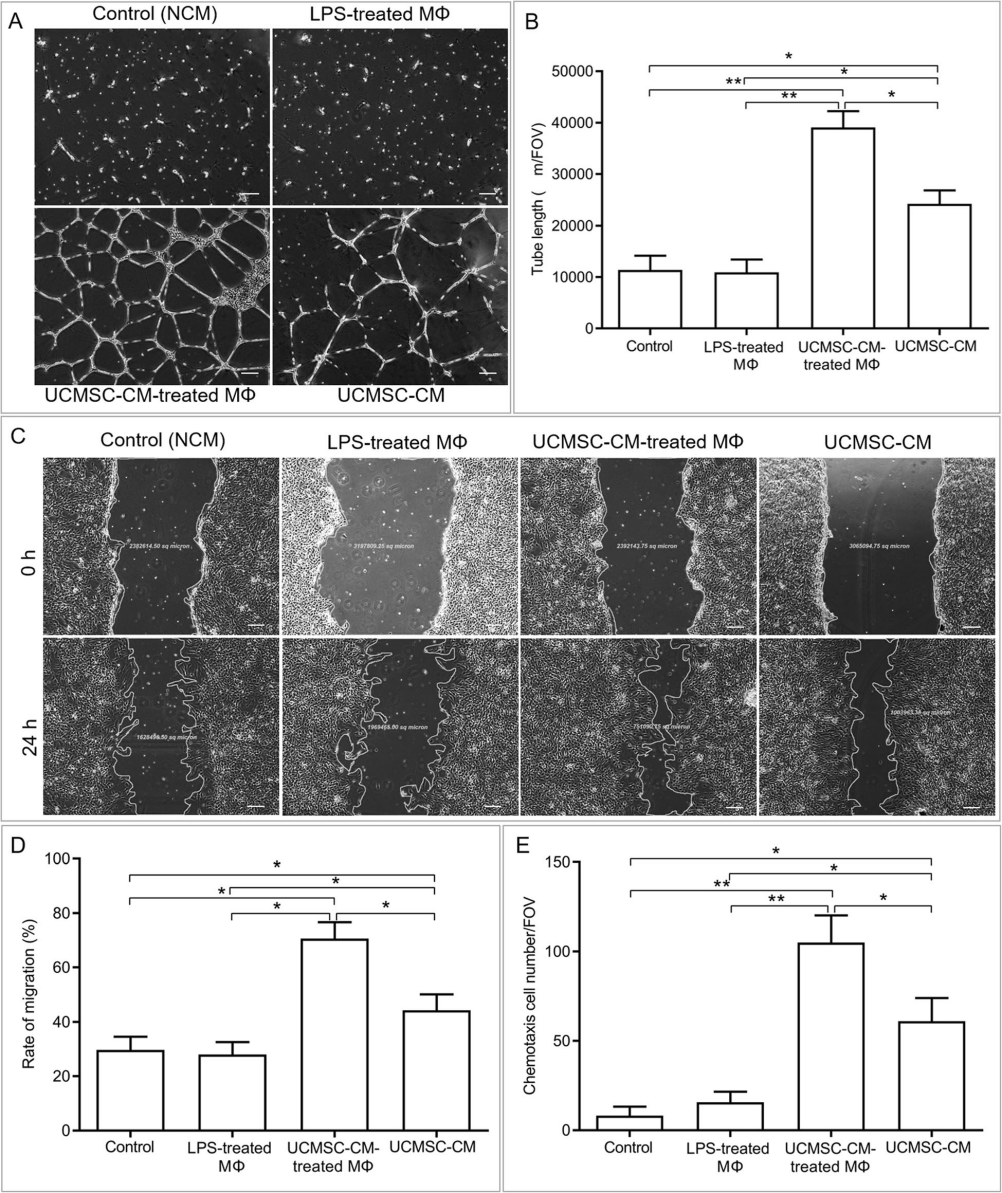
UCMSC-CM restores the dysfunctions of diabetic vascular endothelial cells via M2 macrophages. The functions of high-glucose-induced HUVECs, which were treated with NCM (control) and UCMSC-CM or cocultured with LPS-treated macrophages (MΦ) or UCMSC-CM-treated MΦ, were determined by angiogenesis assays, migration assays, and chemotaxis assays. **a** Representative images of tube network in the angiogenesis assay. Images were taken at 24 h. Scale bar 200 μm. **b** Quantitative analysis of the total tube length using ImageJ software in the angiogenesis assay. **c** Representative images of the migration assay. Images were taken at 0 to 24 h. Scale bar 200 μm. **d** Quantitative analysis of the wound area in the migration assay. The scratch area was measured using the ImageJ software before and after incubation. **e** Quantitative analysis of chemotactic cell number in the chemotaxis assay. The chemotactic cells were fixed with 100% methanol and then stained with DAPI. The numbers of stained nuclei were counted in five high-power fields per well under fluorescence microscopy. *n* = 6, per group. *P < 0.05, **P < 0.01

To investigate the effects of UCMSC- or UCMSC-CM-treated macrophages on the function of diabetic vascular endothelial cells, the diabetic dysfunction of HUVECs was induced by high glucose, and endothelial cell functions, including in vitro angiogenesis assays, migration assays, and chemotaxis assays, were examined when HUVECs were cultured in UCMSC-CM with or without LPS-treated macrophages or UCMSC-CM-treated macrophages. The functions of HUVECs were obviously impaired in the high-glucose medium. As shown in Fig. 5a and b, HUVECs cultured in UCMSC-CM had significantly more tubules per field of view (FOV) than those cultured with LPS-treated macrophages or control medium. The number of tubules derived from HUVECs was further significantly increased by cocultured with UCMSC-CM-treated macrophages when compared with that of HUVECs cultured in UCMSC-CM. Furthermore, the migration assay showed that compared with LPS-treated macrophages or control medium, UCMSC-CM significantly increased the rate of HUVEC migration, and LPS-treated macrophages cultured in UCMSC-CM further markedly enhanced the UCMSC-CM-induced HUVEC migration (Fig. 5c, d). In addition, the chemotaxis assay results revealed that the number of chemotactic HUVECs treated with UCMSC-CM or indirectly cocultured with UCMSC-CM-treated macrophages was greater than that of HUVECs indirectly cocultured with LPS-treated macrophages or in the control medium (Fig. 5e and Additional file 1: Figure S2). The chemotactic ability of HUVECs cultured in UCMSC-CM and indirectly cocultured with UCMSC-CM-treated macrophages was significantly different. Collectively, these results demonstrate that UCMSC-CM-activated macrophages can further enhance the restoration of diabetic endothelial cell function compared with UCMSC-CM.

### PGE2 secreted by UCMSCs is a major mechanism for their recovery effect on diabetic endothelial cell function via the remodeling of the macrophage phenotype

**Fig. 6:**
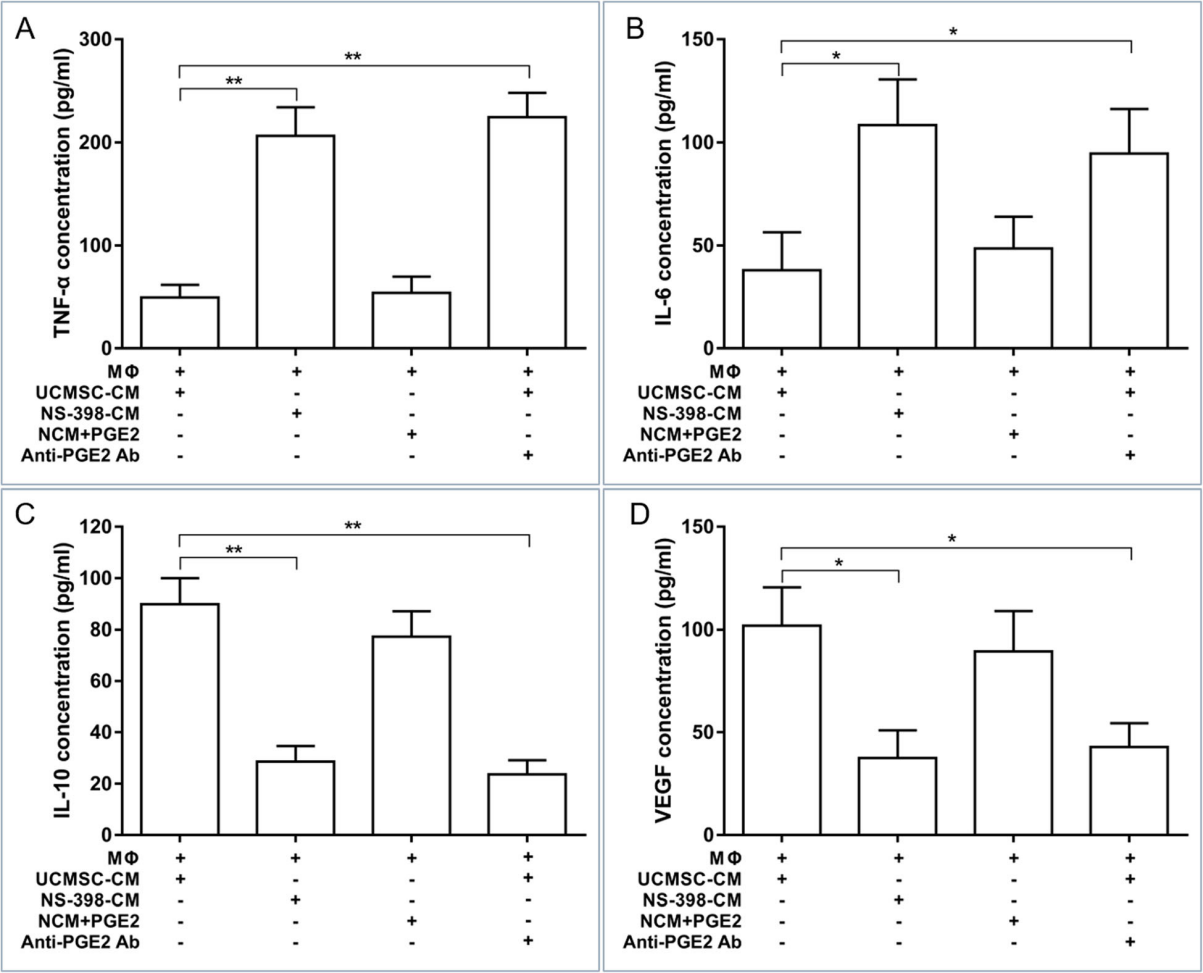
PGE2 mediates the polarization effects of UCMSC-CM on macrophages. LPS-treated macrophages were stimulated with NS-398-CM, a mixture of UCMSC-CM with an anti-PGE2 neutralizing antibody, or NCM plus PGE2. Several cytokines derived from macrophages were detected by ELISA. **a** TNF-α. **b** IL-6. **c** IL-10. **d** VEGF. *n* = 6 per group. *P < 0.05, **P < 0.01

To identify the potential mechanism underlying the UCMSC-mediated rescue of diabetic endothelial cell function, inflammatory factors in UCMSC-CM were examined by ELISA. We found that the concentration of PGE2 secreted by UCMSCs was over 2500 pg/L in the UCMSC-CM, which was abolished by treatment with the PGE2 inhibitors NS-398 and indomethacin (Additional file 1: Figure S3). The addition of PGE2 to the control medium induced the same effects as UCMSC-CM on LPS-treated macrophages, including increasing the levels of IL-10 and VEGF and reducing the levels of TNF-α and IL-6 (Fig. 6). These effects were reversed in NS-398-CM derived from NS-398-treated UCMSCs. When a neutralizing antibody against PGE2 was added to the cultures, UCMSC-CM did not increase the secretion of IL-10 and VEGF and decrease the secretion of TNF-α and IL-6 by LPS-treated macrophages (Fig. 6).

**Fig. 7:**
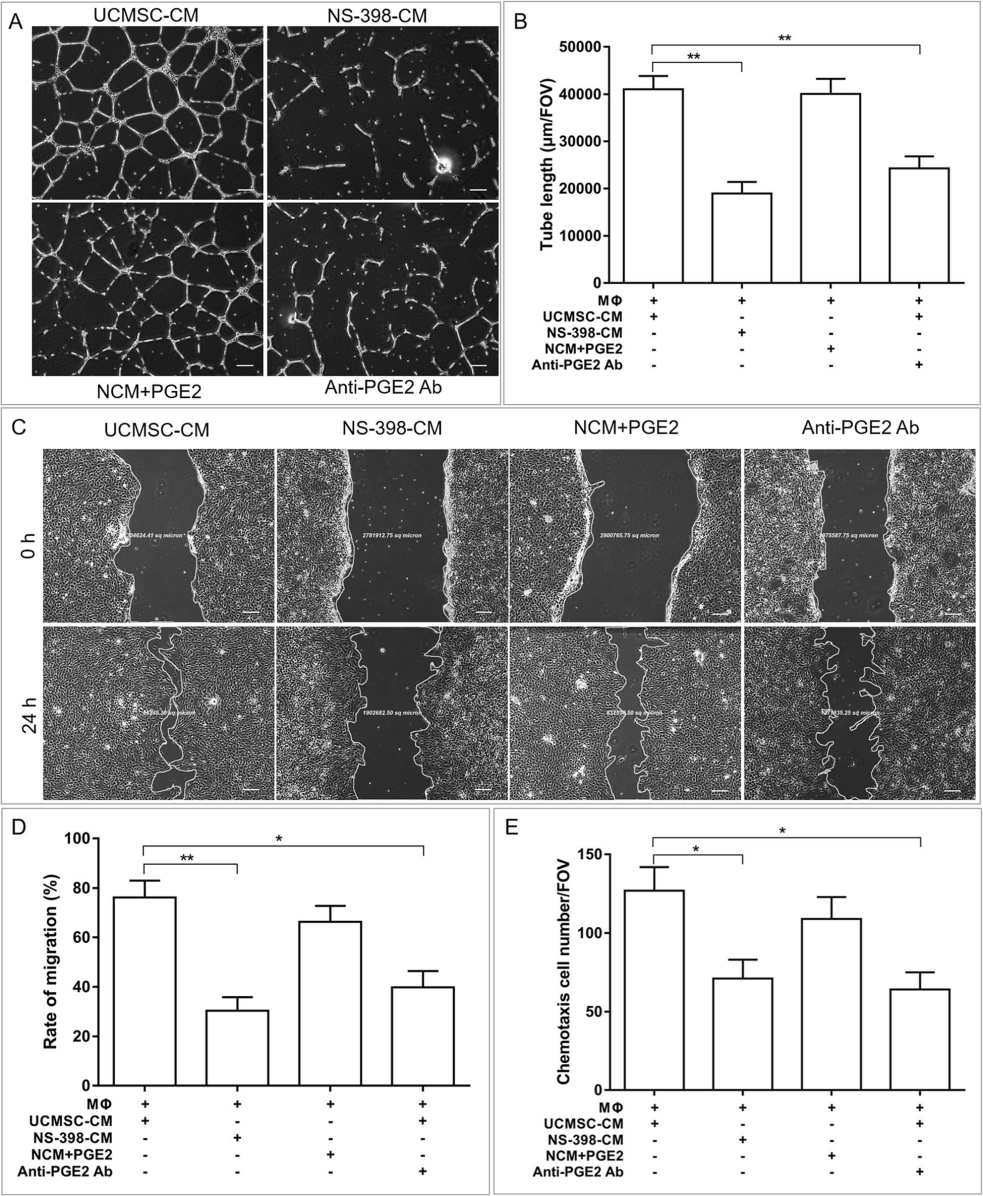
PGE2 plays a role in the UCMSC-CM-induced functional recovery of diabetic vascular endothelial cells via remodeling of the macrophage phenotypes. High-glucose-induced HUVECs, which were indirectly cocultured with macrophages, were treated with NS-398-CM, a mixture of UCMSC-CM with an anti-PGE2 neutralizing antibody, or NCM plus PGE2. The functions of HUVECs were determined by angiogenesis assays, migration assays, and chemotaxis assays. **a** Representative images of the angiogenesis assay. Scale bar 200 μm. **b** Quantitative analysis of the total tube length in the angiogenesis assay. **c** Representative images of the migration assay. Scale bar 200 μm. **d** Quantitative analysis of the wound area in the migration assay. **e** Quantitative analysis of the chemotactic cell number in the chemotaxis assay. *n* = 6 per group. *P < 0.05, **P < 0.01

Furthermore, Fig. 7 shows that the angiogenesis, migration, and chemotaxis abilities of diabetic endothelial cells were also restored by the addition of PGE2 to the control medium with LPS-treated macrophages, which is similar to the effect of UCMSC-CM. The proangiogenic effects of UCMSC-CM-polarized macrophages on diabetic endothelial cells were blunted by treatment with the PGE2 inhibitors NS-398 or the neutralizing antibody against PGE2. Therefore, UCMSC-derived PGE2 plays a key role in the regulation of endothelial cell functions by UCMSCs via macrophages.

## Discussion

**Fig. 8:**
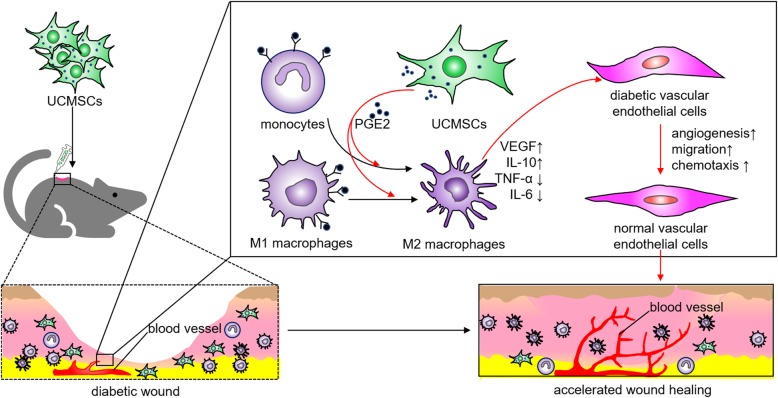
Schematic model of UCMSCs promoting wound healing in diabetic mice. UCMSCs accelerate wound healing and improve angiogenesis in diabetic mice via the remodeling of the macrophage phenotypes. UCMSC-secreted PGE2 plays a crucial role in improving the restoration of vascular endothelial cell dysfunction via the remodeling of macrophage phenotypes, which subsequently improve the local microenvironment of vascular endothelial cells through the secretion of IL-10 and VEGF

Increasing evidence has demonstrated that MSCs derived from different tissues can promote diabetic wound healing [22, 31, 32]. However, a few reports have revealed that UCMSCs may facilitate the repair of diabetic wounds, but the underlying mechanism remains unclear [15, 33]. In the present study, we also confirmed that UCMSCs can accelerate diabetic wound healing, which is consistent with the findings of several previous studies [15, 33]. Furthermore, we found that UCMSC-CM can enhance wound healing in diabetic mice, suggesting that the paracrine effects of UCMSCs play a key role in the promotion of diabetic wound healing. This finding correlates with our previous observations that UCMSCs can rescue acute liver failure in mice by stimulating endogenous cell regeneration through paracrine effects [26]. The present study focused on identifying the underlying mechanism by which transplanted UCMSCs can act as an initiator to trigger endogenous cell-mediated wound healing. Our results indicate that UCMSCs can induce the functional restoration of vascular endothelial cells via remodeling of the macrophage phenotypes, thereby enhancing cutaneous wound healing in diabetic mice (Fig. 8).

Stem cell therapy has become a promising approach in wound healing, and multiple mechanisms contribute to the efficacy of stem cell therapy [34]. In addition to the direct transdifferentiation and regeneration abilities of transplanted cells, trophic effects from cell therapies play a critical role [16]. In this study, we validated the therapeutic potential of UCMSC transplantation for diabetic wound healing. However, we also found that the number of transplanted UCMSCs in wound tissues decreased gradually with time. There is growing evidence that less than 1% of transplanted MSCs can survive for longer than a week following implantation [16, 35]. We further demonstrated that like UCMSC transplantation, the local injection of UCMSC-CM can promote the repair of diabetic wounds, suggesting that UCMSC secretions play an important role in UCMSC transplantation. Therefore, UCMSCs promote diabetic wound healing by regulating endogenous cells or the microenvironment via their paracrine effects.

Several studies have demonstrated that MSCs improve the angiogenesis of diabetic wounds [4, 31]. Our results show that UCMSCs enhance the angiogenesis of diabetic vascular endothelial cells in vivo and in vitro, indicating that UCMSCs may facilitate the functional recovery of diabetic vascular endothelial cells. Impaired angiogenesis is the cause of refractory wounds and is mainly attributed to the dysfunction of vascular endothelial cells caused by a high-glucose-induced wound microenvironment in patients with diabetes mellitus [21]. Macrophages are key regulators of the local inflammatory microenvironment in diabetic wounds [25, 36]. M1 macrophages are characterized by the expression of TNF-α and IL-6, which are generally considered as proinflammatory and inhibit angiogenesis in wound healing [25]. In contrast, M2 macrophages express high levels of arginase-1, IL-10, and VEGF, which are considered as anti-inflammatory and promote angiogenesis in wound healing. Our results demonstrate that UCMSC or UCMSC-CM treatment increases the percentage of endogenous M2 macrophages in the local wounds of diabetic mice. In the local inflammatory microenvironment of diabetic wounds, UCMSC or UCMSC-CM treatment produces high levels of IL-10 and VEGF but low levels of TNF-α and IL-6. In vitro, we found that UCMSCs or UCMSC-CM promote the polarization of macrophages into the anti-inflammatory M2 phenotype characterized by increased levels of the secreted cytokines IL-10 and VEGF and the suppressed production of TNF-α and IL-6. These results are consistent with accumulating evidence showing that MSCs can change endogenous macrophages from the M1 phenotype to the M2 phenotype in diabetic wounds [31, 37]. However, the role of MSC-induced M2 macrophages in promoting angiogenesis in wound healing by MSCs has not been reported. Herein, in vitro assays revealed that UCMSC-induced M2 macrophages can further enhance the action of UCMSCs in promoting the functional recovery of diabetic vascular endothelial cells. Therefore, UCMSCs induce the functional restoration of endogenous vascular endothelial cells via the remodeling of endogenous macrophage phenotypes and enhance cutaneous wound healing in diabetic mice.

The effects of UCMSCs on the polarization of macrophage have been reported in the cell therapy of different diseases [14, 38], but the underlying mechanisms remain unclear. In addition to IL-4 and IL-13, several other soluble factors, including IL-10, GM-CSF, and PGE2, induce the polarization of M2 macrophages under different experimental conditions [25]. In this study, high levels of PGE2 were detected in UCMSC-CM. Previous studies have shown that PGE2 secreted by MSCs derived from other tissues might be responsible for the MSC-induced M2 phenotype of macrophages [39, 40]. We found that the action of the UCMSC-CM-induced M2 phenotype of macrophages was abrogated by the administration of neutralizing antibodies against PGE2 or by inhibiting the secretion of PGE2 from UCMSCs. Furthermore, the proangiogenic effects of UCMSC-CM-polarized macrophages on diabetic vascular endothelial cells were blunted by treatment with NS-398 or a PGE2 neutralizing antibody. Taken together, these findings suggest that UCMSC-derived PGE2 plays a key role in regulating the functional recovery of diabetic vascular endothelial cells by UCMSCs via macrophages.

## Conclusions

In summary, our findings confirm that UCMSCs can accelerate wound healing and improve angiogenesis in diabetic mice. In the UCMSC-dependent promotion of angiogenesis and wound healing, UCMSC-secreted PGE2 plays a crucial role in improving the restoration of vascular endothelial cell dysfunction via the remodeling of macrophage phenotypes, which subsequently improve the local microenvironment of vascular endothelial cells through the secretion of IL-10 and VEGF. Our results suggest that UCMSCs may represent a promising strategy for diabetic wound healing by initiating endogenous cell-mediated tissue repair.

## Supplementary information

**Additional file 1: Table S1.** Quantitative real-time PCR primers used in this study. **Figure S1.** Cell survival and engraftment of PKH26-labeled UCMSCs into wound bed tissues in diabetic mice. **Figure S2.** Representative images of the chemotaxis assays. **Figure S3.** The concentrations of PGE2 were determined by ELISA in the UCMSC-CM and the CM derived from UCMSCs that were cultured in medium with the PGE2 inhibitors NS-398 or indomethacin. **Figure S4.** Representative images of the chemotaxis assays.

## Data Availability

The datasets supporting the conclusions of this article are included within the article and its additional files.

## Abbreviations

CM: Conditioned medium
DAPI: 4,6-Diamidino-2-phenylindole
DMEM: Dulbecco’s modified Eagle’s medium
EGM: Epithelial growth medium
ELISA: Enzyme-linked immunosorbent assay
FBs: Fibroblasts
FBS: Fetal bovine serum
H&E: Hematoxylin and eosin
HUVECs: Human umbilical vein endothelial cells
IL: Interleukin
LPS: Lipopolysaccharide
MSC: Mesenchymal stem cell
NCM: Nonconditioned medium
OCT: Optimal cutting temperature
PGE2: Prostaglandin E2
STZ: Streptozotocin
TNF: Tumor necrosis factor
UCMSC: Umbilical cord-derived matrix stem cell
UCMSC-CM: UCMSC-conditioned medium
VEGF: Vascular endothelial growth factor
