# Supplementary material for: Umbilical cord-matrix stem cells induce the functional restoration of vascular endothelial cells and enhance skin wound healing in diabetic mice via the polarized macrophages
Source: Stem Cell Res Ther. 2020 Jan 28;11:39. doi: 10.1186/s13287-020-1561-x (PMC6986138; doi:10.1186/s13287-020-1561-x)
Supplement: Supplementary file 1 — Additional file 1: Table S1. Quantitative real-time PCR primers used in this study. Figure S1. Cell survival and engraftment of PKH26-labeled UCMSCs into wound bed tissues in diabetic mice. Figure S2. Representative images of the chemotaxis assays. Figure S3. The concentrations of PGE2 were determined by ELISA in the UCMSC-CM and the CM derived from UCMSCs that were cultured in medium with the PGE2 inhibitors NS-398 or indomethacin. Figure S4. Representative images of the chemotaxis assays. [file 13287_2020_1561_MOESM1_ESM.docx]

Supplementary materials

Table S1. Quantitative real-time PCR primers used in this study

| Gene name | Primer |
| --- | --- |
| IL-1β | F: TGACAGTGATGAGAATGACCTGTTC  R: TTGGAAGCAGCCCTTCATCT |
| IL-6 | F: TCGGAGGCTTAATTACACATGTTC  R: TGCCATTGCACAACTCTTTTCT |
| IL-10 | F: CAAAGGACCAGCTGGACAACA  R: GCAACCCAAGTAACCCTTAAAGTC |
| TNF-α | F: GACCCTCACACTCAGATCATCTTCT  R: CCACTTGGTGGTTTGCTACGA |
| TGF-β | F: CGGAGAGCCCTGGATACCA  R: GCCGCACACAGCAGTTCTT |
| VEGF | F: GAGCAGAAGTCCCATGAAGTGAT  R: CAATCGGACGGCAGTAGCTT |
| Arginase-1 | F: CAGAAGAATGGAAGAGTCAG  R: CAGATATGCAGGGAGTCACC |
| GAPDH | F: ATGGTGAAGGTCGGTGTGA  R: CTCCACTTTGCCACTGCAA |


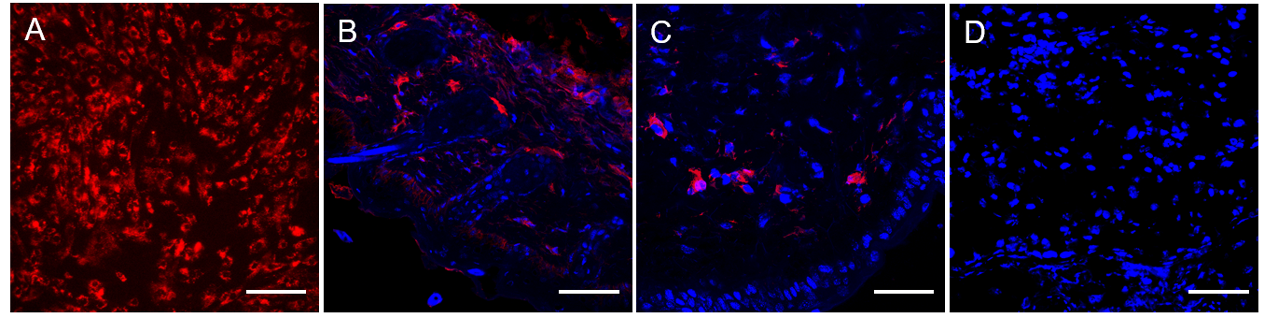


Figure S1. Cell survival and engraftment of PKH26-labeled UCMSCs into wound bed tissues in diabetic mice. (A) UCMSCs prelabeled with PKH26 showing red fluorescence. (B) Representative images of frozen sections of wound bed tissues at 1 day after transplantation. (C) Representative image of frozen sections of wound bed tissues at 7 days after transplantation. (D) Representative images of frozen sections of wound bed tissues at 14 days after transplantation. Nuclei were stained with DAPI (blue). Scale bar: 200 μm.


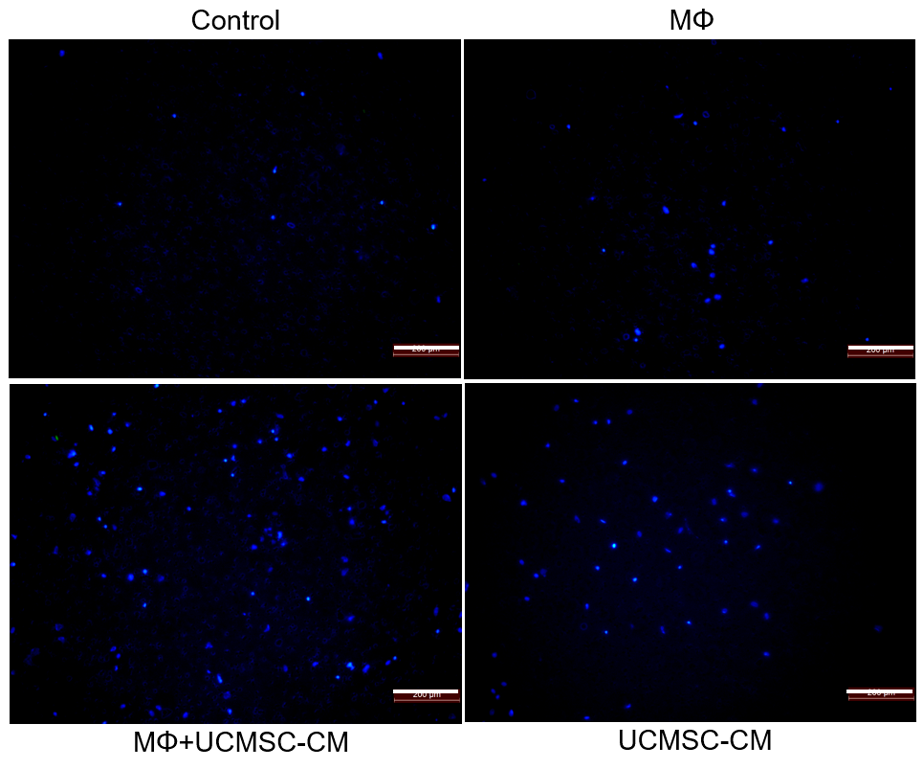


Figure S2. Representative images of the chemotaxis assays. High-glucose-treated HUVECs, which were cultured in the upper chamber of Transwell inserts, were stimulated with NCM (control), UCMSC-CM, or cocultured with LPS-treated macrophages (MΦ) or UCMSC-CM-treated macrophages (MΦ+UCMSC-CM). Cells that migrated to the lower surface of the Transwell membrane were stained with DAPI and photographed under a fluorescence microscope. Scale bar: 200 μm.


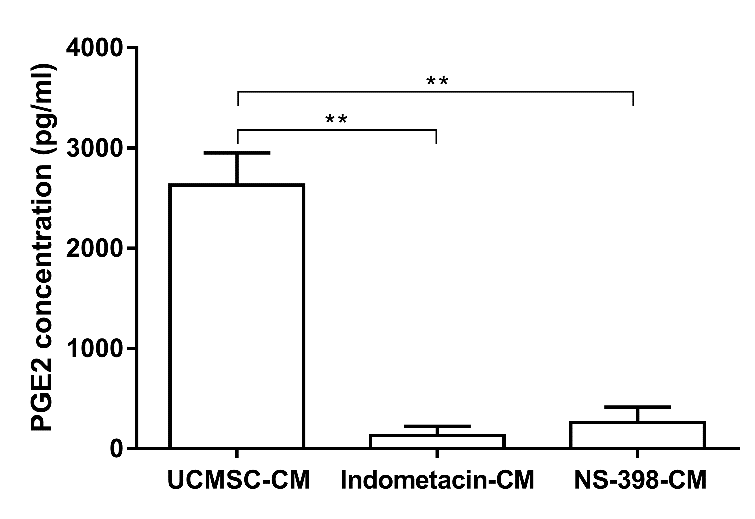


Figure S3. The concentrations of PGE2 were determined by ELISA in the UCMSC-CM and the CM derived from UCMSCs that were cultured in medium with the PGE2 inhibitors NS-398 or indomethacin. n = 6 per group. ** *P* < 0.01.


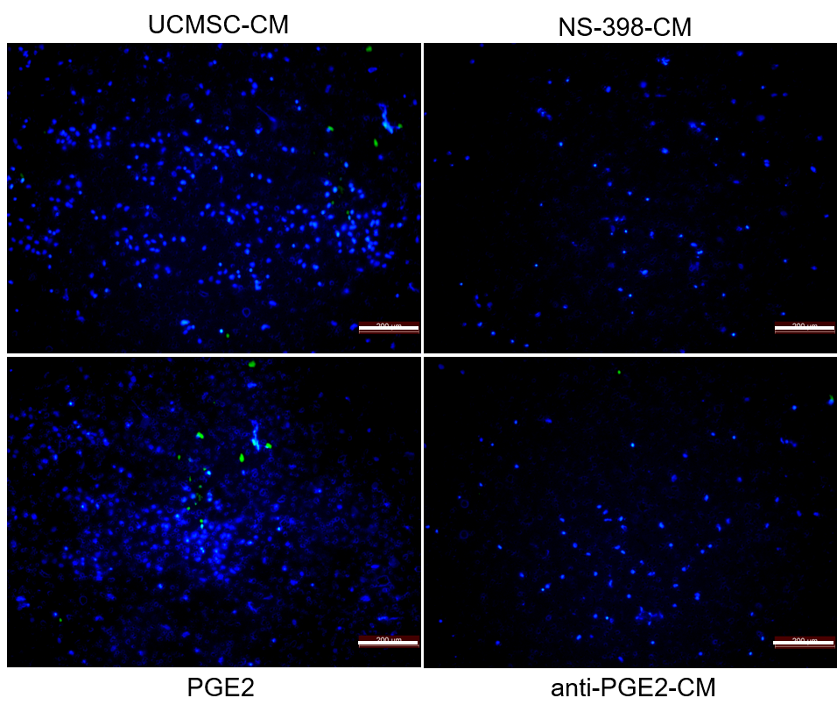


Figure S4. Representative images of the chemotaxis assays. High-glucose-induced HUVECs, which were seeded in the upper chamber of Transwell inserts, were indirectly cocultured with macrophages that were plated in the lower chamber of the Transwell system. This culture system was stimulated with NS-398-CM, the mixture of UCMSC-CM with an anti-PGE2 neutralizing antibody, NCM with PGE2. Cells that migrated to the lower surface of the Transwell membrane were stained with DAPI and photographed under a fluorescence microscope. Scale bar: 200 μm.
